# Prevalence of *Schistosoma* infection and associated factors among pregnant women attending antenatal care at Shewa Robit Health Center, North-Central Ethiopia: a cross-sectional study

**DOI:** 10.1186/s41182-024-00671-8

**Published:** 2024-12-27

**Authors:** Woubshet Zewdie, Getaneh Alemu, Tadesse Hailu

**Affiliations:** 1 Debre Berhan Hospital, Amhara Regional Health Bureau, Debre Berhan, Ethiopia; 2 Department of Medical Laboratory Science, College of Medicine and Health Sciences, Bahir Dar University, P.O. Box 79, Bahir Dar, Ethiopia

**Keywords:** Ethiopia, Pregnant women, Prevalence, *Schistosoma mansoni*

## Abstract

**Background:**

*Schistosoma* spp. and other intestinal parasites are common in Ethiopia. During pregnancy, SCH increases the risk of adverse birth outcomes. However, its epidemiology among pregnant women and awareness level about the disease are not well addressed in Ethiopia. This study was conducted to address this gap.

**Methods:**

A cross-sectional study was conducted from April to June 2023 among 422 pregnant women who attended Shewa Robit Health Center. Pregnant women who came to the health center for antenatal care services were enrolled in the study following systematic random sampling technique. Questionnaire data was collected on socio-demographic characteristics, KAP towards SCH, and associated factors. Stool samples were processed using the Kato–Katz technique, while urine samples were tested with urine test strips for hematuria, and filtration and centrifugation methods for detection of *S. haematobium*. Data were entered and analyzed using SPSS software version 25. Descriptive statistics and logistic regression were performed at a 95% confidence level.

**Results:**

Among 422 pregnant women, 38 (9.0%) were positive for hematuria, but none were infected by *S. haematobium*. *Schistosoma mansoni* was detected in 40 (9.5%; 95% confidence interval (CI): 6.6–12.6) participants. Habit of swimming or bathing (adjusted odds ratio (AOR) = 4.896; 95% CI: 2.193–10.933, *p* < *0.001*) and habit of crossing freshwater on barefoot (AOR = 5.113; 95% CI: 1.171–22.324, *p* = *0.030*) were significantly associated with *S. mansoni* infection. Of the participants, only 74 (17.5%) had previously heard of SCH. Out of 74 aware participants, 49 (66.2%) and 14 (18.9%) were unaware of the causative agent and possibility of a cure for SCH, respectively. Sixty-one (82.4%) were aware that SCH is preventable. Fifty-three (71.6%) and 4 (5.4%) participants believed that SCH is preventable and serious disease, respectively. Eight (10.8%) and 9 (12.2%) participants avoided contact with freshwater and used clean water for drinking and washing, respectively.

**Conclusions:**

There is nearly moderate prevalence of *S. mansoni* infection in the study area. Pregnant women who often had freshwater contact were more likely to contract *S. mansoni*. Most pregnant women in the study area had low KAP levels towards SCH. Therefore, women of reproductive age groups should be the focus of SCH control programs.

## Background

Schistosomiasis (SCH) is one of the neglected tropical diseases, and it is caused by blood flukes of the genus *Schistosoma*. Globally, about 700 million people are at risk of infection, with over 200 million infected and about 200,000 deaths occur annually [1, 2]. The majority, 224 million (90%), of infected people live in sub-Saharan Africa [3]. Among seven species that naturally infect humans, *S. haematobium* and *S. mansoni* are widely spread throughout the world, while all the other species are localized to specific locations [4, 5]. Both *S. haematobium* and *S. mansoni* are endemic in Ethiopia, that 53.3 million people are at risk of infection, while 5.01 million have the disease [6, 7]. *S. mansoni* infection is more prevalent and widespread throughout the country, while *S. haematobum* is limited to the eastern and western lowland borders [8].

Transmission of SCH is dependent on the availability of specific snail hosts and human activities related to freshwater contacts. Individuals with SCH spread the disease by contaminating freshwater sources with their excreta (stool and urine), which contains parasite eggs that hatch in the water to release meracidium, which is infective to snails. After its development and multiplication in the snail, cercarial stages are released and penetrate humans through the intact skin. In humans, the larvae develop into adult *Schistosoma*, and the life cycle continues [9].

Females of reproductive age groups are usually engaged in water-associated domestic activities like washing clothes or utensils and fetching water from freshwater sources for domestic consumption; hence they are at risk of *Schistosoma* infection. *Schistosoma* affects approximately 40 million reproductive-age women globally, including 10 million African women [10]. If women are infected during pregnancy, more severe morbidities and increased vulnerability to other diseases are common, partly due to their weakened immune response [11]. Despite this, most previous epidemiological studies and intervention programs in Ethiopia target children, systematically leaving pregnant women untreated [12].

Schistosomiasis signs and symptoms are commonly set by the body’s response to the larvae (cercariae) and the eggs of the parasite rather than the worms themselves. Skin rashes, fever, and abdominal pain are manifested in the acute stage, while the chronic stage of the disease may result in severe anemia, liver, intestinal and urogenital diseases. Other complications, like adverse birth outcomes, are seen if the infection happens during pregnancy. Symptoms are highly dependent on the species of the parasite and the host immune status [13].

Microscopy after urine sedimentation or filtration and chemical reagent strips are the most commonly used procedures for diagnosing genitourinary SCH. The Kato–Katz technique is the ‘Gold Standard’ test recommended by the World Health Organization (WHO) for the diagnosis of intestinal SCH [4].

There are limited data on *Schistosoma* infection among pregnant women in Ethiopia. Furthermore, there is limited knowledge about factors associated with *Schistosoma* infection and knowledge, attitudes and practice (KAP) towards SCH among pregnant women is unknown. Hence, the aim of this study was to assess the prevalence of SCH, KAP level and associated factors among pregnant women in Kewot district, Shewa Robit Health Center, where both *S. mansoni* and *S. haematobium* are co-endemic.

## Methods

### Study design, area and period

**Fig. 1:**
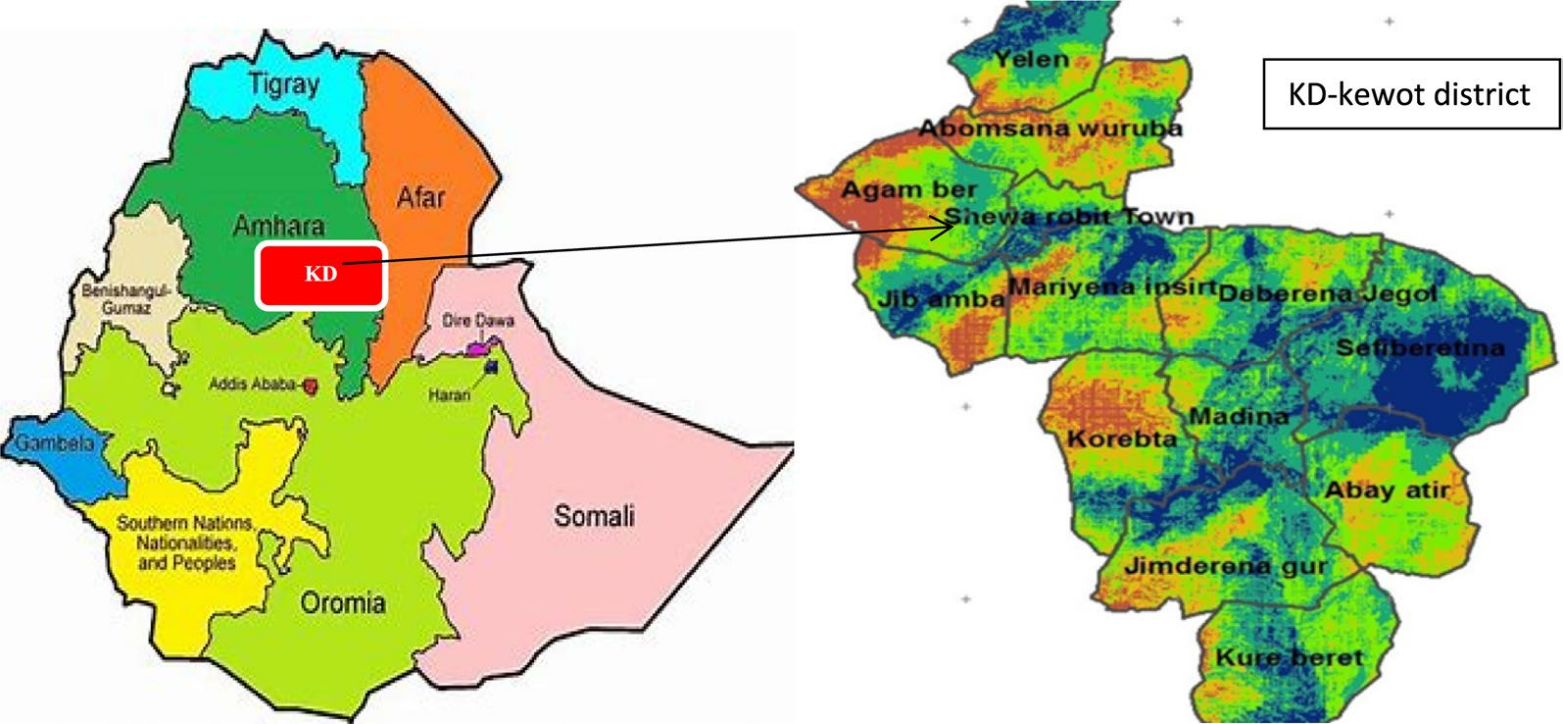
Map of the study area

A health-facility-based cross-sectional study was conducted from April to June 2023 to assess the prevalence of *schistosoma* infection and associated factors among pregnant women attending Shewa Robit Health Center, kewot district, north-central Ethiopia. Kewot district is located at latitude and longitude coordinates of 10°00′N, and 39°54′E in North Shewa zone, Amhara Region (Fig. 1). It has 18 Kebeles and one city administration. One River that flows throughout the year crosses the district and there are many tributaries which dry up in the dry season. Shewa Robit is the capital city of Kewot district. This district has an average altitude of 1280 m above sea level, a mean annual rainfall of 1330 mm, a mean annual temperature ranging from 22 to 30 °C, and a humidity of 58% [14].

### Sample size determination and sampling technique

Sample size was determined using a single population proportion formula depending on the following assumptions: 50% prevalence (since there was no previous study in the area), 95% confidence level, 5% margin of error and 10% non-respondent rate. The total sample size, including 10% for the non-respondents rate was 422. Shewa Robit Health Center was purposively selected among the four health centers found in Kewot district. Data on the number of pregnant women who visited the health center from April to June in the previous year (2022) was used to calculate the sampling interval, which was 714. Hence, selection of study participants was made by systematic random sampling technique with a sampling interval (K) of 2 (714/422 = 1.7~2). The study participants were accessed at the antenatal care unit. The first participant was selected by lottery method, and then every other pregnant woman was enrolled in the study.

Women who were confirmed to be pregnant either by human chorionic gonadotropin hormone test or ultrasound, permanently lived (at least for 6 months) in Kewot district, and gave consent to participate in the study were included. Pregnant women who had taken anti-helminthic drugs within the last 3 months before data collection, who came for delivery, or those severely ill and unable to respond to the research questions or unable to provide stool and urine samples were excluded from the study.

## Data collection

### Questionnaire-based data

A structured questionnaire was prepared in English and translated to the local language (Amharic) to collect data on socio-demographic characteristics; water, sanitation and hygiene (WASH); and KAP towards SCH. The questionnaire was administered through a face-to-face interview by midwives.

### Urine sample collection and *S. haematobium* examination

Urine samples were collected between 10 AM and 2 PM, and processed in the Shewa Robit Health Center laboratory. After proper instruction, each woman was given a labeled graduated plastic urine container to collect about 30 ml of random midstream urine. Urine samples were analyzed using reagent strips to detect hematuria. The test strip was completely immersed in a well-mixed urine sample, placed on a paper towel for 1–2 min, and interpreted based on the dipstick’s analysis chart [15]. Participants only underwent urine filtration if the urine strip test revealed hematuria. In short, a polycarbonate or nylon fiber filter (pore size 12–20 µm) was placed in the filter holder. The urine sample was well mixed, and 10 ml was taken into a syringe. The filter holder was attached, and the urine in the syringe was drained to pass through the filter. Then, the filter was removed using forceps and was put on a microscope slide. The entire filter was examined after staining with Lugol’s iodine, and eggs were identified based on their morphology [15, 16]. A remaining 10 ml of urine was centrifuged at 1000 rpm for 5 min. The supernatant was discarded, and a drop of Lugol’s iodine was added to a drop of the sediment. The sediment was examined under the 10× objective to detect ova of *S. haematobium*.

### Stool sample collection and *S. mansoni* examination

At the time of urine collection, participants were provided a stool cup with an applicator stick to collect approximately 5 g of fresh stool sample. The stool sample was processed and examined by the Kato–Katz technique. About 2–3 g of the fresh sample was pressed through a mesh screen to get ride of large particles. After being sieved, the stool was moved to the template, which was then put on a slide until the template hole which could hold 41.7 mg of stool—was filled. Then, the template was removed, and the stool sample was covered and pressed with cellophane, which had been previously submerged overnight with glycerol–malachite green solution. Within 30–60 min, all fields of the Kato–Katz smear were inspected for ova of hookworms, and the examination was repeated after 24 h for detection and quantitation of *S. mansoni* eggs. Eggs of intestinal parasites were identified based on their morphological differences [16]. The fecal egg count, expressed as eggs per gram of stool (epg), was calculated by multiplying the egg count by 24. The epg was used to classify *S. mansoni* infections as light (epg < 100), moderate (epg: 100–399) and heavy (epg ≥ 400) [11, 17].

### Data quality assurance

Training was given to data collectors about the sample collection procedures and the questionnaire. A pre-test was done on 5% of the sample size (21 participants) before the actual data collection began. Stool and urine sample collection and investigation were made in accordance with standard operating procedures. Each Kato–Katz and urine smear was read by two medical laboratory technologists and a third senior medical laboratory technologist resolved any discrepancies.

### Data analysis

All data collection forms were checked for completeness and reliability before entry into the software. Data entered and analyzed using SPSS software version 25. Descriptive statistics was used to compute prevalence and proportion of *Schistsoma* infections and KAP of participants. Factors associated with *S. mansoni* infection were first analyzed by univariate logistic regression. Then, to control the possible confounding factors, variables with *P* value ≤ 0.2 were adjusted by multivariate logistic regression. Variables with *p* < *0.05* at a 95% confidence level were considered statistically significant.

## Results

### Socio-demographic characteristics of study participants

In the present study, all enrolled pregnant women completed the questionnaires and provided stool and urine samples, resulting in a 100% response rate. The participants’ ages ranged from 18 to 46 years, with a mean ± standard deviation (SD) and median age of 28.05 ± 3.9 and 28, respectively. The most frequent age was 30; 62 participants (14.7%) were 30 years. The majority of the participants were within the age group of 26–33 (71.1%), and urban residents were 387 (91.7%). More than half, 225 (53.3%), of the participants were housewives, while only 40 (9.5%) attended higher education. Among 40 (9.5%) participants infected by *S. mansoni*, 17 (42.5%), 20 (50.0%) and 3 (7.5%) had light, moderate and heavy intensity infections, respectively (Table 1).

**Table 1:** Socio-demographic characteristics and *S. mansoni* infection among pregnant women attending antenatal care unit in Shewa Robit Health Center, North-Central Ethiopia, 2023 (*N* = 422)

Variable	Category	Number (%)	*S. mansoni* infected *N* (%)	Infection intensity *N*		
				Light	Moderate	Heavy
Age group (in years)	15–25	96 (22.7)	9 (9.4)	6	2	1
	26–33	300 (71.1)	29 (9.7)	11	16	2
	34–49	26 (6.2)	2 (7.7)	0	2	0
Marital status	Married	420 (99.5)	39 (9.3)	16	20	3
	Single	2 (0.5)	1	1	0	0
Residence	Urban	387 (91.7)	31 (8.0)	15	13	3
	Rural	35 (8.3)	9 (25.7)	2	7	0
Educational status	No formal education	78 (18.5)	3 (3.8)	0	2	1
	Primary	192 (45.5)	15 (7.8)	6	9	0
	Secondary	112 (26.5)	15 (13.4)	7	7	1
	College and above	40 (9.5)	7 (17.5)	4	2	1
Occupation	Farmer	23 (5.5)	6 (26.1)	2	4	0
	Day labourer	101 (23.9)	11 (10.9)	6	4	1
	Private/salaried	73 (17.3)	4 (5.5)	1	2	1
	House wife	225 (53.3)	19 (8.4)	8	10	1
**Total**		422 (100)	40 (9.5)	17 (42.5)	20 (50.0)	3 (7.5)

### Prevalence of *Schistosoma*

Among 422 participating pregnant women, urine strip test results revealed that 38 (9.0%) were positive for hematuria, while the rest, 384 (91.0%), were negative. Microscopic examination by both urine centrifugation and filtration techniques shows that none of the participants were infected with *S. haematobium* or any other parasite of the urogenital tract.

Stool examination revealed that 72 (17.1%) participants were infected by at least a single intestinal parasite species. Sixty-six (15.6%) participants were infected with a single parasite species, while 6 (1.4%) were co-infected with two parasite species. *Schistosoma mansoni* was the most common parasite detected in 40 (9.5%; 95% CI: 6.6–12.6) participants, followed by hook worm, which was detected in 20 (4.7%) women (Table 2).

**Table 2:** Prevalence of intestinal parasitic infections among pregnant women attending antenatal care unit in Shewa Robit Health Center, North-Central Ethiopia, 2023 (*N* = 422)

Parasite spp detected	Frequency (%)
*Schistosoma mansoni*	40 (9.5)
Hookworm	20 (4.7)
*Ascaris lumbriciodes*	12 (2.8)
*Trichuris trichiura*	1(0.2)
*Enterobius vermicularis*	3 (0.7)
*Hymenolepis nana*	1 (0.2)
*Taenia* species	1 (0.2)
**Overall**	78 (18.5)

The minimum, maximum, mean ± SD and median epgs of *S. mansoni* were 48, 960, 161.4 ± 161.3 and 120, respectively. Among 40 *S. mansoni*-infected women, 36 were mon-infected while 3 and 1 women were co-infected with hook worm and *A. lumbricoides*, respectively.

### Factors associated with *Schistosoma mansoni* infection

Among candidate variables for multivariate analysis, the habit of swimming or bathing in surface water (adjusted odds ratio (AOR) = 4.896; 95% confidence interval (CI): 2.193–10.933, *p* < *0.001*) and the habit of crossing surface water on barefoot (AOR = 5.113; 95% CI: 1.171–22.324, *p* = *0.030*) were significantly associated with *S. mansoni* infection (Table 3).

**Table 3:** Logistic regression of factors associated with *S. mansoni* infection among pregnant women attending antenatal care unit in Shewa Robit Health Center, North-Central Ethiopia, 2023 (*N* = 422)

Variable	Category	Number examined (%)	Positive (%)	COR (95% CI)	*P *value	AOR (95% CI)	*P *value
Age group (in years)	15–25	96 (22.7)	9 (9.4)	1.241 (0.251–6.133)	0.791		
	26–33	300 (71.1)	29 (9.7)	1.284 (0.289–5.712)	0.743		
	34–49	26 (6.2)	2 (7.7)	1			
Residence	Rural	35 (8.3)	9 (25.7)	3.975 (1.712–9.228)	0.001	4.250 (0.795–22.732)	0.091
	Urban	387 (91.7)	31 (8.0)	1			
Educational status	No formal education	78 (18.5)	3 (3.8)	0.189 (0.046–0.775)	0.021	0.388 (0.073–2.076)	0.269
	Primary	192 (45.5)	15 (7.8)	0.400 (0.151–1.055)	0.064	1.187 (0.358–3.935)	0.779
	Secondary	112 (26.5)	15 (13.4)	0.729 (0.274–1.943)	0.527	1.324 (0.436–4.020)	0.620
	College and above	40 (9.5)	7 (17.5)	1			
Occupation	Private/salaried	73 (17.3)	4 (5.5)	1			
	Farmer	23 (5.5)	6 (26.1)	6.088 (1.544–24.006)	0.010	0.172 (0.015–2.003)	0.160
	Day labourer	101 (23.9)	11 (10.9)	2.108 (0.644–6.907)	0.218	0.771 (0.186–3.202)	0.721
	House wife	225 (53.3)	19 (8.4)	1.591 (0.523–4.838)	0.413	0.478 (0.122–1.872)	0.289
Toilet utilization	Yes	412 (97.6)	37 (9.3)	1			
	No	10 (2.4)	3 (30.0)	4.344 (1.078–17.510)	0.039	2.540 (0.450–14.332)	0.291
Swimming or bathing in surface water	Yes	78 (18.5)	20 (25.6)	5.586 (2.830–11.025)	0.000	4.896 (2.193–10.933)	<0.001
	No	344 (81.5)	20 (5.8)	1			
Cross surface water barefoot	Yes	83(19.7)	18 (21.7)	3.990 (2.026–7.858)	0.000	5.113 (1.171–22.324)	0.030
	No	339 (80.3)	22 (6.5)	1			
Wash clothes in surface water	Yes	83(19.7)	16 (19.3)	3.134 (1.579–6.220)	0.001	0.625 (0.140–2.801)	0.540
	No	339 (80.3)	24 (7.1)	1			
Participate in irrigation	Yes	48 (11.4)	8 (16.7)	2.137 (0.922–4.957)	0.077	0.366 (0.104–1.287)	0.117
	No	374 (88.6)	32 (8.6)	1			
Water source	Pipe	418 (99.1)	39 (9.3)	1			
	Surface water	4 (0.9)	1 (25.0)	3.239 (0.329–31.894)	0.314		
Heard about schistosomiasis	Yes	74 (17.5)	5 (6.8)	1			
	No	348 (82.5)	38 (10.9)	1.543 (0.583–4.081)	0.382		

*AOR* adjusted odds ratio, *COR *crude odds ratio, *CI* confidence interval

### Knowledge, attitude and practice towards schistosomiasis

#### Knowledge about schistosomiasis

Among a total of 422 participants, only 74 (17.5%) had ever heard about SCH. The primary source of information was schools that 59 (79.7%) participants out of 74 responded as they got the information from schools. Among participants who have heard of SCH, 49 (66.2%) did not know the causative agent; 30 (40.5%) did not know about the involvement of snails in the disease transmission; 13 (17.6%) did not know any of human activities attributive to *Schistosoma* transmission; 24 (32.4%) did not mention at least one sign and symptom; and 14 (18.9%) did not know whether schistosomiasis is curable or not (Table 4).

**Table 4:** Knowledge about schistosomiasis among pregnant women attending antenatal care unit in Shewa Robit Health Center, North-Central Ethiopia, 2023

Knowledge area	Responses	Number	%
Have you ever heard about schistosomiasis or bilharzia? (*N* = 422)	Yes	74	17.5
	No	348	82.5
If ‘yes’, what was your source of information? (*N* = 74)	School	59	79.7
	Health institutions/campaigns	15	20.3
What is the causative agent of schistosomiasis?	Worm	25	33.8
	Don’t know	49	66.2
Do snails involve in the transmission of schistosomiasis?	Yes	44	59.5
	Don’t know	30	40.5
Which activities are attributive for schistosomiasis transmission?	Defecating or urinating near fresh water	4	5.4
	Swimming in fresh water	42	56.8
	Washing or bathing in fresh water	14	18.9
	Walking barefoot in water	1	1.4
	Don’t know	13	17.6
How do we know a person has schistosomiasis?	By signs and symptoms	1	1.4
	By laboratory diagnosis	60	81.1
	Don’t know	13	17.5
What are the signs and symptoms of schistosomiasis?	Abdominal pain	49	66.2
	Belly enlargement	1	1.4
	Don’t know	24	32.4
Can schistosomiasis be cured?	Yes	60	81.1
	Don’t know	14	18.9
Is schistosomiasis preventable disease?	Yes	61	82.4
	No	2	2.7
	Don’t know	11	14.9
If ‘yes’, how can you prevent it? (*N* = 61)	Avoid contact with freshwater bodies	45	73.8
	Use clean water for drinking and washing	7	11.5
	Wear protective gear during water contact	3	4.9
	Avoid open defecation	9	14.8

#### Attitude towards schistosomiasis

Among 74 pregnant women who were aware of schistosomiasis, 53 (71.6%) believed that the disease can be prevented, while the remaining 21 (28.4%) believed that SCH is not a preventable disease. The majority (82.4%) of aware participants knew that schistosomiasis is prevalent in their residence area (Kewot district). However, out of 74 participants, only 4 (5.4%) believed that SCH is a serious disease (Table 5).

**Table 5:** Attitude towards schistosomiasis among pregnant women attending antenatal care unit in Shewa Robit Health Center, North-Central Ethiopia, 2023 (*N* = 74)

Attitude area	Responses	Number	%
Do you believe that schistosomiasis can be prevented?	Yes	53	71.6
	No	21	28.4
Do you believe Schistosomiasis is prevalent in your area?	Yes	61	82.4
	No	1	1.4
	Not sure	12	16.2
Do you believe schistosomiasis is a serious disease?	Yes	4	5.4
	No	41	55.4
	Not sure	29	39.2

#### Practices for prevention of schistosomiasis

For the sake of preventing schistosoma infection, 8 (10.8%) participants responded that they avoid contact with fresh water, and 9 (12.2%) use clean water for drinking and washing (Table 6).

**Table 6:** Practice for schistosomiasis prevention among pregnant women attending antenatal care unit in Shewa Robit Health Center, North-Central Ethiopia, 2023 (*N* = 74)

	Practice	Number	%
Good practices for prevention of schistosomiasis	Avoid contact with fresh water	8	10.8
	Use clean water for drinking and washing	9	12.2
	Wear protective gear in infected water	1	1.4
	Do nothing	51	68.9

## Discussion

Schistosomiasis control and prevention programs in Ethiopia primarily targeted school-aged children, despite other segments of the population, like pregnant women and people whose occupation is closely linked to freshwater also being at risk of infection. In pregnant women, apart from the risk of infection, the morbidity is more serious than in other population groups, as SCH contributes to adverse birth outcomes. According to the previous national mapping data, both *S. mansoni* and *S. haematobium* are reported from Kewot district among school-aged children (Source: Amhara Regional Health Bureau). However, *S. haematobium* was with zero prevalence in the present study. This might be due to the focal distribution of the parasite even within a district. Moreover, infections might be missed as *S. haematobium* has intermittent egg output, and only a single urine sample was examined. Presence of blood in urine (hematuria) might be due to other causes like urinary tract infection, kidney or bladder cancer and inherited hematological disorders, among others.

The prevalence of *S. mansoni* in the present study lies on the upper limit of the low prevalence range (1– < 10%) [4]. This was higher than previous findings of 3.0% in ten Carebbean countries [18], 0.83% in Nigeria [19], 2.9% in Felegehiwot referral hospital, Ethiopia [20], and 3.2% in Yifag health center, Ethiopia [21]. Variations in the local transmission of *S. mansoni* and the sample size might contribute to the difference in prevalence. For instance, studies from Nigeria and Yifag enrolled a lower sample size of 120 [19] and 280 [21], respectively, which decreases the results of the findings.

On the other side, the prevalence of *S. mansoni* in the present study was lower as compared to previous findings of 63.5% in Tanzania [22], 13.0% in Sudan [23], 28.0% in Cameroon [11], 17.4% in Mecha [24], 33.0% in Shewa Robit town [25], and 13.7% in Hawassa [26]. Differences in the number of stool samples collected and examined might contribute to this. For example, while a study from Tanzania collected and examined two stool samples from each participant [22], this present study conducted one stool examination per participant. Variations in diagnostic methods used also bring differences in prevalence. The study from Camerroon used Kato–Katz and formol–ether concentration [11], while the study from Hawassa used direct wet mount and formol–ether concentration [26], but only Kato–Katz was used in the present study. It is known that the use of combined diagnostic methods increases the detection rate, and hence the prevalence. The year of data collection might also be another factor, as it is directly linked to the duration of the implementation of SCH control programs. Similarly, differences in endemicity of *Schistosoma* parasites and implementation of prevention and control programs are possible factors for variability in *S. mansoni* prevalence across countries and localities within a country.

Certain socio-demographic characteristics, activities or habits that expose people to freshwater are thought to predispose people to *Schistosoma* infection [27]. The odds of *S. mansoni* infection was 4.896 among pregnant women with the habit of swimming or bathing in freshwater compared to those who do not swim or bath. This is justifiable, because the infective cercaria swims in freshwater and infects people when they are having direct contact with the infested water. Likewise, pregnant women who frequently crossed surface water barefoot were also 5.113 times at higher risk of infection compared to their counterparts. People cross rivers during travel to markets, agricultural fields or any other service area. They might do this every day or repeatedly, that increases their exposure to *Schistosoma* cercaria.

The risk of *Schistosoma* infection was associated with certain occupations like participation in fishing and irrigation [27]. Accordingly, a previous study from Nigeria reported that occupation is significantly associated with *Schistosoma* infection (*p* = *0.022*) [28]. However, there was no significance found in the present study (*p* > *0.05*). The majority of participants were with occupations related to freshwater contact, which made them almost equally at occupational risk. Lack of knowledge about schistosomiasis was significantly associated in a previous study from Cameroon [29]. In the present study, the awareness level of participants was not associated with *Schistosoma* infection, as aware pregnant women in the present study knew only the name of the disease but had no adequate knowledge. Knowing the name of the disease without knowledge about the transmission, clinical outcome and prevention methods does not enable people to protect themselves from the infection.

The low proportion of pregnant women who had ever heard of the disease indicated that pregnant women were not targeted for control programs in the study area. A higher percentage (23.8%) of women were aware of the disease, according to a previous study in Dembia, Ethiopia [30]. Even the majority of aware participants get the information from schools, either while they were at school or through schoolchildren. On top of this, the majority of aware participants knew only the name of the disease but had no adequate knowledge regarding the causative agent, route and modes of transmission, clinical importance and prevention methods. Because pregnant women had no adequate knowledge about the clinical outcomes and complications during pregnancy, the majority of them did not believe it is a serious disease, or they are not sure whether it is serious or not. This might hinder the majority of aware women from practicing any of the preventive methods, despite 82.4% knowing that schistosomiasis is a preventable disease and 71.6% knew that the disease is prevalent in their residence area.

## Limitations

Data for the present study was collected from a health institution, excluding pregnant women who did not attend antenatal care. Moreover, data were collected in a low *Schistosoma* transmission season; hence, the findings might underestimate the prevalence in the study area. A single stool and urine sample were collected and the stool was examined only with Kato–Katz due to limited logistics.

## Conclusions

There is nearly moderate prevalence of *S. mansoni* infection in the study area among pregnant women. No *S. haematobium* infection was reported in the present study despite hematuria being detected in 38 participants. Habit of swimming or bathing in surface water and the habit of crossing surface water on barefoot were significantly associated with *S. mansoni* infection. The majority of pregnant women in the study area have low KAP levels towards schistosomiasis. Therefore, schistosomiasis control and prevention programs and the health education packages should target women of reproductive age at the antenatal care unit.

## Data Availability

No data sets were generated or analysed during the current study.

## Abbreviations

AOR: Adjusted Odds Ratio
CI: Confidence Interval
epg: Eggs per gram
KAP: Knowledge Attitudes and Practice
KK: Kato Katz
SCH: Schistosomiasis
WASH: Water, Sanitation and Hygiene
WHO: World Health Organization
